# Two organelle RNA recognition motif proteins affect distinct sets of RNA editing sites in the *Arabidopsis thaliana* plastid

**DOI:** 10.1002/pld3.213

**Published:** 2020-04-06

**Authors:** Audrey M. Searing, Manasa B. Satyanarayan, James P. O′Donnell, Yan Lu

**Affiliations:** ^1^ Department of Biological Sciences Western Michigan University Kalamazoo MI USA

**Keywords:** multiple organellar RNA editing factors, organelle RNA recognition motif proteins, organelle zinc finger proteins, pentatricopeptide repeat proteins, plastid RNA editing, RNA editing interacting proteins

## Abstract

Plastid and mitochondrial RNAs in vascular plants are subjected to cytidine‐to‐uridine editing. The model plant species *Arabidopsis thaliana* (Arabidopsis) has two nuclear‐encoded plastid‐targeted organelle RNA recognition motif (ORRM) proteins: ORRM1 and ORRM6. In the *orrm1* mutant, 21 plastid RNA editing sites were affected but none are essential to photosynthesis. In the *orrm6* mutants, two plastid RNA editing sites were affected: *psbF*‐C77 and *accD*‐C794. Because *psbF* encodes the β subunit of cytochrome *b*_559_ in photosystem II, which is essential to photosynthesis, the *orrm6* mutants were much smaller than the wild type. In addition, the *orrm6* mutants had pale green leaves and reduced photosynthetic efficiency. To investigate the functional relationship between ORRM1 and ORRM6, we generated *orrm1 orrm6* double homozygous mutants. Morphological and physiological analyses showed that the *orrm1 orrm6* double mutants had a smaller plant size, reduced chlorophyll contents, and decreased photosynthetic efficiency, similar to the *orrm6* single mutants. Although the *orrm1 orrm6* double mutants adopted the phenotype of the *orrm6* single mutants, the total number of plastid RNA editing sites affected in the *orrm1 orrm6* double mutants was the sum of the sites affected in the *orrm1* and *orrm6* single mutants. These data suggest that ORRM1 and ORRM6 are in charge of distinct sets of plastid RNA editing sites and that simultaneous mutations in *ORRM1* and *ORRM6* genes do not cause additional reduction in editing extent at other plastid RNA editing sites.

## 1 INTRODUCTION

RNA editing is a post‐transcriptional process through which discrete changes are introduced to RNA sequences. In plants, RNA editing is restricted to plastids and mitochondria and is viewed as a correction mechanism to compensate for mutations in the haploid organelle genomes (Lu, 2018; Shi, Hanson, & Bentolila, 2017a; Sun, Bentolila, & Hanson, 2016; Takenaka, Zehrmann, Verbitskiy, Hartel, & Brennicke, 2013). A common type of plant organelle (plastid and mitochondrion) RNA editing is cytidine‐to‐uridine (C‐to‐U) deamination. Different plant species may have different plastid and mitochondrial C‐to‐U RNA editing sites (Bentolila, Oh, Hanson, & Bukowski, 2013; Chateigner‐Boutin & Small, 2007; Ruwe, Castandet, Schmitz‐Linneweber, & Stern, 2013). In mammalian species, C‐to‐U RNA editing is catalyzed by the APOBEC‐type cytidine deaminase (Xu & Messing, 2006). However, in plant plastids and mitochondria, C‐to‐U RNA editing is carried out by the RNA editing complex, which contains at least four types of editing factors: pentatricopeptide repeat (PPR) and PPR‐related proteins, RNA editing interacting proteins/multiple organellar RNA editing factors (RIPs/MORFs), organelle zinc finger (OZ) proteins, and organelle RNA recognition motif (ORRM) proteins (Lu, 2018; Shi, Hanson, et al., 2017; Sun et al., 2016; Takenaka et al., 2013).

PPR proteins are ubiquitously present in eukaryotes (Barkan & Small, 2014; Fujii & Small, 2011). Although most eukaryotes contain less than a dozen PPR proteins, land plants contain > 400 PPR proteins and all of them are predicted or have been shown to be localized to the plastids or mitochondria (Lurin et al., 2004). PPR‐E‐DYW‐type PPRs, which contain a PPR domain, an E (extension) domain, and a DYW domain, and PPR‐E‐type PPRs, which contain a PPR domain and an E domain, were found to be involved in C‐to‐U RNA editing in land plant plastids and mitochondria (Chateigner‐Boutin et al., 2013; Lu, 2018; Okuda et al., 2009; Okuda, Myouga, Motohashi, Shinozaki, & Shikanai, 2007; Sun et al., 2016). DYW1, a PPR‐related protein, was also found to participate in this process (Boussardon et al., 2012, 2014). The PPR domain has the ability to bind to RNAs in a sequence‐specific manner (Okuda, Nakamura, Sugita, Shimizu, & Shikanai, 2006; Okuda & Shikanai, 2012; Schallenberg‐Rüdinger, Kindgren, Zehrmann, Small, & Knoop, 2013; Tasaki, Hattori, & Sugita, 2010; Williams‐Carrier, Kroeger, & Barkan, 2008). The DYW domain has a [HXE(X)_n_CXXC] motif, which is homologous to the signature zinc finger motif in classic cytidine deaminases (Boussardon et al., 2014; Faivre‐Nitschke, Grienenberger, & Gualberto, 1999). Therefore, PPR‐ and PPR‐related proteins are prime candidates for C‐to‐U deamination in land plant organelles (Lu, 2018). In line with the sequence‐specific binding between a PPR protein and the corresponding RNA target, PPR proteins are rarely found to interact with each other. However, a number of PPR proteins have been found to interact with other RNA editing factors, including RIPs/MORFs (Bentolila et al., 2012; Takenaka et al., 2012; Wagoner, Sun, Lin, & Hanson, 2015; Zhang et al., 2014), ORRM1 (Sun et al., 2013), and OZ1 (Sun et al., 2015).

RIP/MORF proteins contain conserved RIP/MORF domains and are only present in the plastids and mitochondria of flowering plants (Lu, 2018; Takenaka et al., 2013). The *Arabidopsis thaliana* (Arabidopsis) nuclear genome encodes nine functional RIPs/MORFs (Bentolila et al., 2012; Takenaka et al., 2012). Among them, RIP2/MORF2 and RIP9/MORF9 are plastid‐targeted, RIP1/MORF8 is dually targeted to plastids and mitochondria, and the other six are mitochondrion‐targeted (Bentolila et al., 2012; Takenaka et al., 2012). RIPs/MORFs do not contain any RNA‐binding domains; however, RIPs/MORFs may form homodimers or heterodimers (Takenaka et al., 2012; Zehrmann et al., 2015). Furthermore, RIPs/MORFs were found to interact with other types of RNA editing factors, including PPRs (Bentolila et al., 2012; Takenaka et al., 2012; Zhang et al., 2014), OZ1 (Sun et al., 2015), and ORRMs (Hackett et al., 2017; Shi, Germain, Hanson, & Bentolila, 2016b; Shi, Hanson, & Bentolila, 2015). Therefore, it was proposed that RIPs/MORFs act as a scaffold, bridging different components of the RNA editing complex together (Lu, 2018).

OZ proteins contain Ran‐binding‐protein2 (RanBP2, CXXCX_10_CXXC)‐type zinc finger domains and are found in many land plants (Sun et al., 2015). RanBP2‐type zinc fingers are capable of binding to RNAs in a sequence‐specific manner (Nguyen et al., 2011). The Arabidopsis nuclear genome encodes four OZ proteins: OZ1, OZ2, OZ3, and OZ4, which contain two, two, three, and four RanBP2‐type zinc fingers, respectively (Sun et al., 2015). OZ3 is predicted to be targeted to the mitochondrion; OZ1, OZ2, and OZ4 are predicted to be plastid‐targeted. The plastid localization of OZ1 has been experimentally confirmed (Sun et al., 2015). OZ1 has been found to interact with itself and other plastid RNA editing factors, such as RIP1/MORF8, ORRM1, and ORRM6 (Hackett et al., 2017; Sun et al., 2015).

ORRM proteins are a subfamily of organelle‐localized RNA recognition motif (RRM) proteins (Lu, 2018; Shi, Hanson, et al., 2017). RRM proteins are capable of binding to RNAs and are present in viruses, bacteria, and eukaryotes (Maris, Dominguez, & Allain, 2005). The Arabidopsis nuclear genome encodes six ORRM proteins: ORRM1 and ORRM6 are plastid‐targeted; ORRM2, ORRM3, ORRM4, and ORRM5 are mitochondrion‐targeted (Lu, 2018; Shi, Hanson, et al., 2017). ORRM1 contains an ORRM domain at the C‐terminus and two truncated RIP/MORF domains at the N‐terminus; ORRM2 and ORRM6 contain an ORRM domain at the C‐terminus; ORRM3, ORRM4, and ORRM5 contain an N‐terminal ORRM domain and a C‐terminal glycine‐rich domain (Hackett et al., 2017; Shi, Bentolila, & Hanson, 2016a; Shi, Castandet, Germain, Hanson, & Bentolila, 2017b; Shi, Germain, et al., 2016; Shi et al., 2015; Sun et al., 2013).

Recombinant ORRM1 protein was found to bind preferentially to ORRM1‐dependent RNA editing sites in vitro, via the ORRM domain (Sun et al., 2013). The duplicated RIP/MORF moiety of ORRM1 was found to be required for the interaction between ORRM1 and selective plastid‐targeted PPR proteins, such as CHLORORESPIRATORY REDUCTION28 (CRR28) and ORGANELLE TRANSCRIPT PROCESSING82 (OTP82) (Sun et al., 2013). ORRM1 was also found to interact with other plastid‐targeted editing factors, including RIP1/MORF8, RIP2/MORF2, and OZ1 (Sun et al., 2015). The loss‐of‐function *orrm1‐1* Arabidopsis mutant displayed near‐complete loss of editing at 12 plastid RNA editing sites and substantial reduction in editing extent at nine plastid RNA editing sites (Sun et al., 2013). Although a large number of plastid RNA editing sites were affected, the *orrm1‐1* mutant did not display any phenotypic defect under standard growth conditions (Sun et al., 2013).

Unlike the *orrm1‐1* mutant, the loss‐of‐function *orrm6‐1* and *orrm6‐2* Arabidopsis mutants showed near‐complete loss of editing at *psbF*‐C77 and substantial reduction in editing extent at *accD*‐C794 (Hackett & Lu, 2017; Hackett et al., 2017). *psbF* encodes the β subunit of cytochrome *b*_559_, an essential component of photosystem II (PSII). Consequently, the *orrm6‐1* and *orrm6‐2* mutants displayed reduced PSII photochemical efficiency, small and pale green leaves, and stunted growth (Hackett & Lu, 2017; Hackett et al., 2017). Consistent with the plastid RNA editing pattern in the *orrm6‐1* and *orrm6‐2* mutants, recombinant ORRM6 protein was found to bind preferentially to synthetic RNAs flanking 40 nucleotides upstream and 19 nucleotides downstream of *psbF*‐C77 and *accD*‐C794 (Hackett et al., 2017). Furthermore, ORRM6 was found to interact with itself and other plastid RNA editing factors, including RIP1/MORF8, RIP2/MORF2, RIP9/MORF9, and OZ1 (Hackett et al., 2017).

As mentioned above, the *orrm1‐1* single mutant showed substantial reduction in editing extent at nine plastid RNA editing sites (Sun et al., 2013). This begs the question whether other plastid‐targeted ORRM or ORRM‐like protein(s), such as ORRM6, is responsible for residual editing at these nine plastid RNA editing sites in the *orrm1‐1* single mutant. The *orrm6* single mutants displayed substantial reduction in editing extent at *accD*‐C794 (Hackett & Lu, 2017; Hackett et al., 2017). This raises the question whether other plastid‐targeted ORRM or ORRM‐like protein(s), such as ORRM1, is responsible for residual editing at *accD*‐C794 in the *orrm6* single mutants. Furthermore, recombinant ORRM6 protein showed some binding activity toward the synthetic RNA flanking *psbE*‐C214, a plastid RNA editing site not affected by the loss‐of‐function mutations in the *ORRM6* gene (Hackett et al., 2017). This made us consider whether ORRM6 could function at additional plastid RNA editing sites that are not identified by loss‐of‐function mutations in the *ORRM6* gene. To investigate the functional relationship between the two plastid‐targeted ORRM proteins and explore the possible existence of ORRM‐like proteins in the Arabidopsis plastid, we generated *orrm1 orrm6* double homozygous Arabidopsis mutants, examined their plastid RNA editing pattern, perform a series of morphological and physiological analyses, and compared them with the wild type and the single mutants. The results showed that ORRM1 and ORRM6 are in charge of distinct sets of plastid RNA editing sites.

## 2 MATERIALS AND METHODS

### 2.1 Plant materials and growth conditions

*Arabidopsis thaliana *(Arabidopsis) T‐DNA insertion lines *orrm1‐1* (SALK_072648), *orrm6‐1* (SAIL_763_A05), and *orrm6‐2* (WiscDsLox485‐488P23) in the Columbia ecotype were obtained from the Arabidopsis Biological Resource Center (Sessions et al., 2002; Woody, Austin‐Phillips, Amasino, & Krysan, 2007). Homozygosity of the *orrm1‐1*, *orrm6‐1*, and *orrm6‐2* single mutants and the *orrm1‐1 orrm6‐1* and *orrm1‐1 orrm6‐2* double mutants was confirmed by PCR, using the Phire Plant Direct PCR kit (Thermo Scientific) and genotyping primers listed in Table S1. Plants were grown in a growth chamber (Percival Scientific) on a 12‐hr light/12‐hr dark photoperiod. The light intensity was 150 μmol photons m^−2^ s^−1^, the temperature was 22°C, and the relative humidity was 50%. Unless otherwise stated, plants used for photographing, pigment extraction and measurements, chlorophyll fluorescence, as well as leaf total RNA extraction and subsequent RT‐PCR and Sanger sequencing, were four weeks old.

### 2.2 Measurements of pigment contents

Total chlorophyll and carotenoid were extracted from rosette leaves with 80% acetone in 2.5 mM HEPES‐KOH, pH7.5, and the amounts (mg) of chlorophyll *a* and *b* and carotenoid per gram of fresh tissues were measured on a BioMate 3S UV‐Visible spectrophotometer (Thermo Scientific) at four wavelengths: 470, 646, 654, and 663 nm (Wellburn, 1994).

### 2.3 Chlorophyll fluorescence measurements

Chlorophyll fluorescence parameter *F*_v_ */F*_m_ (maximum photochemical efficiency of PSII) was determined on dark‐adapted plants at room temperature with the MAXI version of the IMAGING‐PAM M‐Series chlorophyll fluorescence system (Heinz Walz GmbH), as described previously (Lu, Hall, & Last, 2011). *F_v_*/*F_m_* is calculated as follows: *F_v_/F_m_* = (*F*_m_ − *F*_o_)/*F*_m_, where *F*_v_, *F*_m_, and *F*_o_ are variable, maximal, and minimal fluorescence of dark‐adapted leaves, respectively (Nath, O'Donnell, & Lu, 2017; Nath, Wessendorf, & Lu, 2016).

### 2.4 Analysis of plastid RNA editing by Sanger sequencing

Total RNA was extracted from Arabidopsis rosette leaves using the RNeasy plant mini kit (QIAGEN), digested with the RNase‐free DNase I (QIAGEN), and reverse‐transcribed with random primers (Promega) and Moloney murine leukemia virus reverse transcriptase (Promega) to generate the mRNA:cDNA hybrids, as described previously (Clark & Lu, 2015). The transcript regions encompassing the Arabidopsis plastid RNA editing sites were amplified using Phusion High‐Fidelity DNA Polymerase (New England Biolabs) and PCR amplification/Sanger sequencing primers listed in Table S1. The resulting PCR products were sequenced at the Michigan State University Genomics Facility, using the Sanger method and the PCR amplification/Sanger sequencing primers listed in Table S1.

### 2.5 Accession numbers

Sequence data of related genes/proteins can be found in the GenBank/EMBL databases under the following accession numbers: ORRM1, At3g20930; ORRM6, At1g73530.

## 3 RESULTS

### 3.1 Identification of *orrm1 orrm6* double homozygous mutants

To explore the functional relationship between ORRM1 and ORRM6, we created *orrm1 orrm6* double homozygous Arabidopsis mutants. The *orrm1‐1* (Sun et al., 2013) and *orrm6‐1* and *orrm6‐2* (Hackett & Lu, 2017; Hackett et al., 2017) homozygous mutants were crossed, and the resulting F_2_ populations were screened for *orrm1‐1 orrm6‐1* and *orrm1‐1 orrm6‐2* double homozygous mutants. Genotyping was performed by amplifying DNA directly from two‐week‐old plants, using the Phire Plant Direct PCR kit (Thermo Scientific). The *orrm1‐1*, *orrm6‐1*, and *orrm6‐2* alleles were genotyped with primer combinations SALK_072648LP + SALK_072648RP and SALK_072648RP + LBa1, SAIL_763_A05LP + SAIL_763_A05RP and SAIL_763_A05RP + LB3, and WiscDsLox485‐488P23LP + WiscDsLox485‐488P23RP and WiscDsLox485‐488P23LP + p745, respectively (Table S1). Self‐fertilized F_3_ seeds harvested from double homozygous F_2_ plants were used to grow plants for downstream analyses.

### 3.2 The *orrm1 orrm6* double mutants adopted the phenotype of the *orrm6* single mutants

As reported in a previous study (Sun et al., 2013), the *orrm1‐1* mutant did not show any phenotypic defect, presumably because none of the plastid RNA transcripts affected in the *orrm1‐1* mutant is essential. The *orrm1‐1* mutant was actually slightly bigger than the Columbia wild type. However, it is not clear whether loss‐of‐function mutation in the *ORRM1* gene causes changes in pigment contents and photosynthetic efficiency. The *orrm6‐1* and *orrm6‐2* mutants were substantially smaller than the wild type, and they displayed reduced PSII photochemical efficiency, small and pale green leaves, and stunted growth (Hackett et al., 2017), presumably because *psbF*, one of the two plastid RNA transcripts affected in the *orrm6* mutants, encodes an essential PSII subunit. To examine whether loss‐of‐function mutation in the *ORRM1* gene causes changes in fresh weights, leaf numbers, pigment contents, and photosynthetic efficiency and whether simultaneous loss‐of‐function mutations in *ORRM1* and *ORRM6* genes result in additive effects, we compared phenotypes, measured fresh weights of the above‐ground portion of the plants, counted rosette leaf numbers, determined pigment contents, and measured photosynthetic parameters in four‐week‐old wild type, *orrm1* and *orrm6* single and double mutants.

**Figure 1:**
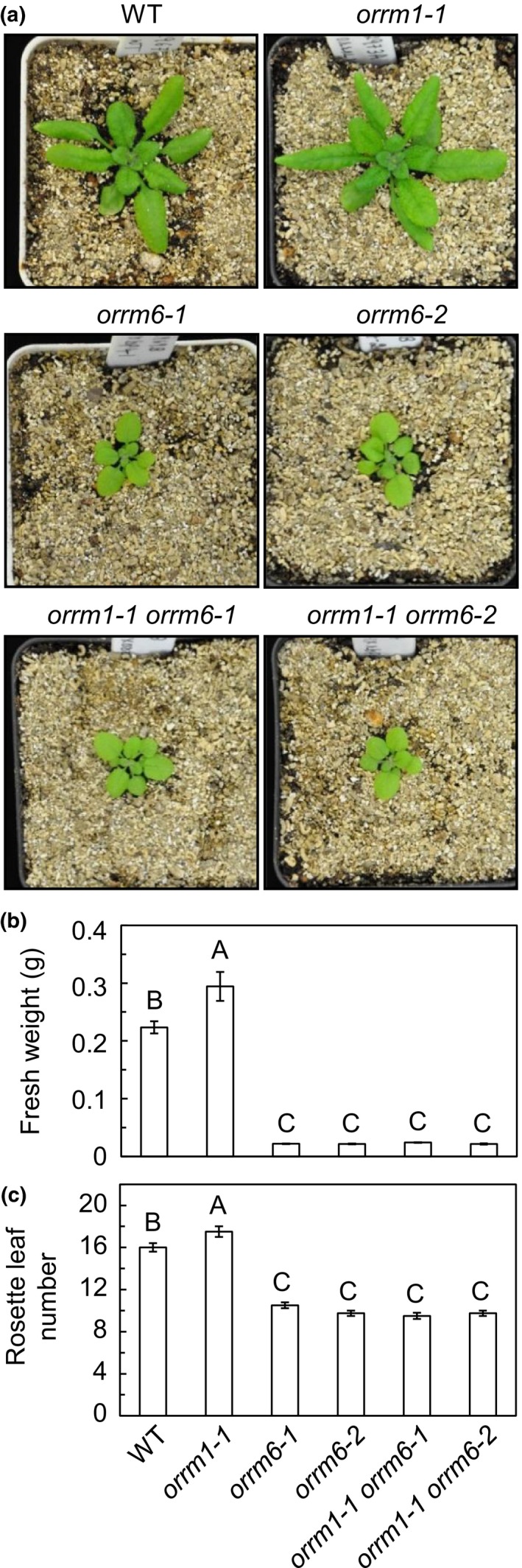
Morphology of 4‐week‐old wild type, *orrm1* and *orrm6* single mutants, and *orrm1 orrm6* double mutants. (a) Images of 4‐week‐old plants. (b) Fresh weights of 4‐week‐old plants. Data are presented as means ± SE (*n* = 6). (c) Rosette leaf numbers of four‐week‐old plants. Data are presented as means ± SE (*n* = 4). Values not connected by the same uppercase letters are significantly different (Student's *t* test, *p* < .05). Plants used for photographing, fresh weights, leaf number counting, pigment extraction, chlorophyll fluorescence analysis, and RNA extraction were grown on a 12‐hr light/12‐hr dark photoperiod with an irradiance of 150 μmol photons m^−2^ s^−1^ during the light period

The *orrm1‐1* single mutant was indeed larger than the Columbia wild type (Figure 1a). The phenotype of the *orrm1‐1 orrm6‐1* and *orrm1‐1 orrm6‐1* double mutants largely resembled the *orrm6‐1* and *orrm6‐2* single mutants: They were much smaller than the wild type, with small and pale green leaves and retarded growth (Figure 1a). The *orrm1‐1* single mutant had a significantly heavier fresh weight (Figure 1b) and a significantly larger rosette leaf number (Figure 1c) than the wild type grown at the same time under the same conditions. This suggests that the *orrm1‐1* single mutant is truly bigger and possibly more advanced in its development than the wild type. The *orrm1‐1 orrm6‐1* and *orrm1‐1 orrm6‐2* double mutants had statistically similar fresh weights (Figure 1b) and statistically similar leaf numbers (Figure 1c) as the *orrm6‐1* and *orrm6‐2* single mutants. This suggests that the *orrm1 orrm6* double mutants are indeed phenotypically similar to the *orrm6* single mutants.

**Figure 2:**
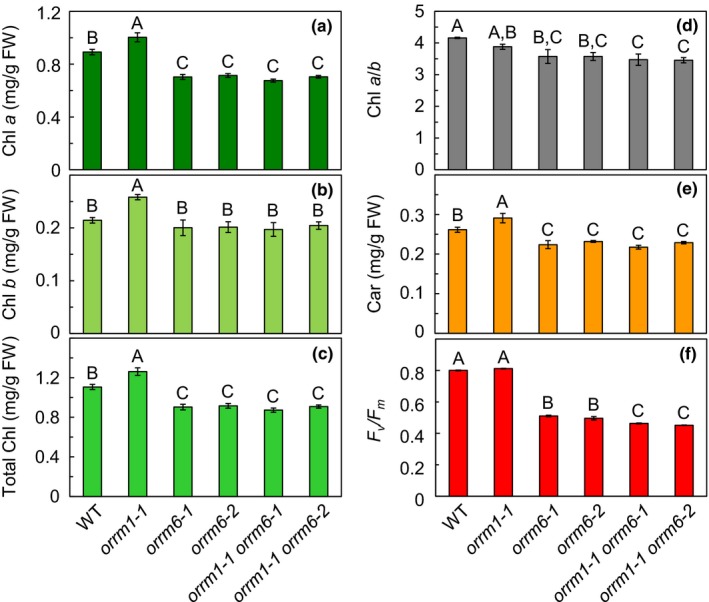
Pigment contents and chlorophyll fluorescence of 4‐week‐old plants. (a–e) Chlorophyll *a* (a), chlorophyll *b* (b), total chlorophyll (c), chlorophyll *a*/*b* ratio (d), carotenoid (e), and *F_v_*/*F_m_* (f) of 4‐week‐old plants. Chlorophyll and carotenoid were extracted and determined as described by Wellburn (1994). Measurements of chlorophyll fluorescence parameters were performed with the IMAGING‐PAM M‐Series chlorophyll fluorescence system (Heinz Waltz) on dark‐adapted plants. Data are presented as means ± SE (*n* = 5 for pigment contents and *n* = 4 for chlorophyll fluorescence parameters). Values not connected by the same uppercase letters are significantly different (Student's *t* test, *p* < .05). Plants used for pigment extraction and fluorescence analysis were grown on a 12‐hr light/12‐hr dark photoperiod with an irradiance of 150 μmol photons/m^−2^ s^−1^ during the light period. Chl, chlorophyll. Car, carotenoid. FW, fresh weight. WT, wild type

The contents of chlorophyll *a*, chlorophyll *b*, and total chlorophyll in the *orrm1‐1* mutant were 12%, 20%, and 14% higher than those in the wild type, respectively (Figure 2a–c). Due to the differential increase in the contents of chlorophyll *a* and *b*, the chlorophyll *a*/*b* ratio in the *orrm1‐1* mutant was slightly lower than that in the wild type (Figure 2d). The contents of chlorophyll *a*, chlorophyll *b*, and total chlorophyll in the *orrm6‐1* and *orrm6‐2* mutants were approximately 21%, 6%, and 18% lower than those in the wild type, respectively (Figure 2a–c). Due to the differential decrease in the contents of chlorophyll *a* and *b*, the chlorophyll *a*/*b* ratio in the *orrm6‐1* and *orrm6‐2* mutants was 14% lower than that in the wild type (Figure 2d). The chlorophyll *a*, chlorophyll *b*, and total chlorophyll contents and the chlorophyll *a*/*b* ratio in the *orrm1‐1 orrm6‐1* and *orrm1‐1 orrm6‐2* double mutants resembled the *orrm6‐1* and *orrm6‐2* single mutants: They were significantly lower than that in the wild type but statistically similar to those in the *orrm6* single mutants (Figure 2a–d). These results are consistent with the pale green pigmentation observed in the *orrm6* single mutants and the *orrm1 orrm6* double mutants (Figure 1a).

The carotenoid level in the *orrm1‐1* mutant was 11% higher than that in the wild type; the carotenoid level in the *orrm6‐1* and *orrm6‐2* mutants was 14% and 11% lower than that in the wild type (Figure 2e). The level of carotenoid in the *orrm1‐1 orrm6‐1* and *orrm1‐1 orrm6‐2* double mutants resembled the *orrm6‐1* and *orrm6‐2* single mutants: It was significantly lower than that in the wild type but statistically similar to that in the *orrm6* single mutants (Figure 2e).

To assess whether the *orrm1* and *orrm6* single and double mutants have defects in PSII, we determined *F*_v_ */F*_m_, an indicator of the maximum photochemical efficiency of PSII (Baker, Harbinson, & Kramer, 2007). *F*_v_ */F*_m_ in the *orrm1‐1* mutant was statistically similar to that in the wild type; however, *F*_v_ */F*_m_ in the *orrm6‐1* and *orrm6‐2* mutants was 36%–38% lower than that in the wild type (Figure 2f). *F*_v_ */F*_m_ in the *orrm1‐1 orrm6‐1* and *orrm1‐1 orrm6‐2* double mutants was 42%–44% lower than that in the wild type and 9% lower than that in the corresponding *orrm6* single mutants (Figure 2e). The substantial decreases in *F*_v_ */F*_m_ in the *orrm6* single mutants and the *orrm1 orrm6* double mutants are consistent with significant reductions of editing extent at the *psbF*‐C77 site in these mutants (Hackett & Lu, 2017; Hackett et al., 2017).

Taken together, the *orrm1 orrm6* double mutants had similar levels of chlorophyll *a*, chlorophyll *b*, total chlorophyll, chlorophyll *a*/*b* ratio, carotenoid, and *F*_v_ */F*_m_ as the *orrm6* single mutants, suggesting that the *orrm1 orrm6* double mutants adopted the phenotype of the *orrm6* single mutants.

### 3.3 The total number of plastid RNA editing sites affected in the double mutants was the sum of sites affected in the single mutants

**Figure 3:**
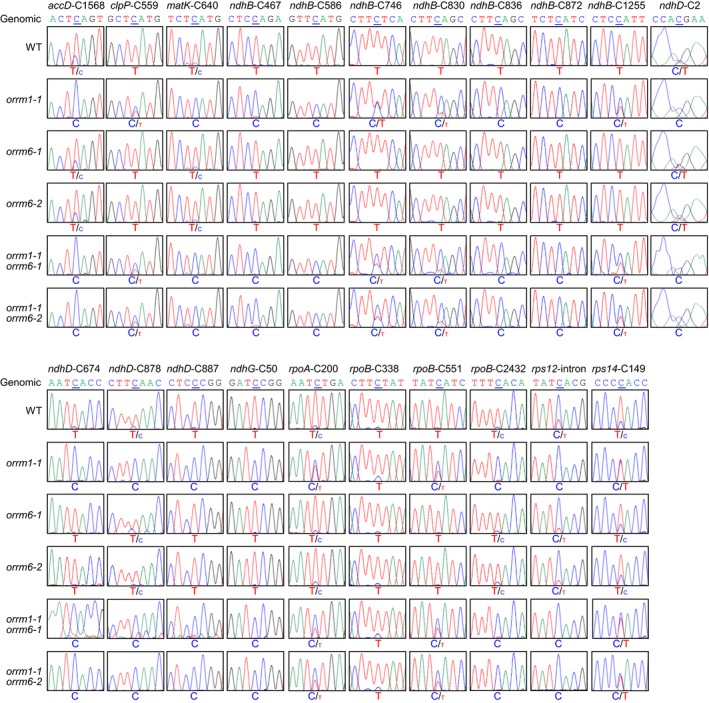
Sanger sequencing of 21 plastid RNA editing sites affected by the loss‐of‐function mutation in *ORRM1*. RT‐PCR products surrounding the editing sites were directly sequenced. The seven‐nucleotide sequences encompassing the cytidine target (underlined) were shown. The corresponding genomic sequences of these sites were displayed as controls

We examined the editing patterns of 34 validated plastid RNA editing sites (Table S2) (Chateigner‐Boutin & Small, 2007) with high‐fidelity PCR amplification and Sanger sequencing, using primers designed previously (Table S1) (Cai et al., 2009; Hackett et al., 2017). The *orrm1‐1* single mutant was previously found to show near‐complete loss of editing at 12 plastid RNA editing sites: *accD*‐C1568, *matK*‐C640, *ndhB*‐C467, *ndhB*‐C586, *ndhB*‐C836, *ndhB*‐C872, *ndhD*‐C674, *ndhD*‐C878, *ndhD*‐C887, *ndhG*‐C50, *rpoB*‐C2432, and *rps12*‐intron (Sun et al., 2013). In this study, these 12 plastid RNA editing sites displayed similar loss of editing in the *orrm1‐1* single mutant and the *orrm1‐1 orrm6‐1* and *orrm1‐1 orrm6‐2* double mutants but were unchanged in the *orrm6‐1* and *orrm6‐2* single mutants (Figure 3). In addition to these 12 sites, the *orrm1‐1* single mutant showed reduction in editing extent at nine plastid RNA editing sites: *clpP*‐C559, *ndhB*‐C746, *ndhB*‐C830, *ndhB*‐C1255, *ndhD*‐C2, *rpoA*‐C200, *rpoB*‐C338, *rpoB*‐C551, and *rps14*‐C149 (Sun et al., 2013). In this study, these nine plastid RNA editing sites displayed similar reduction in editing extent in the *orrm1‐1* single mutant and the *orrm1‐1 orrm6‐1* and *orrm1‐1 orrm6‐2* double mutants but was unchanged in the *orrm6‐1* and *orrm6‐2* single mutants (Figure 3).

**Figure 4:**
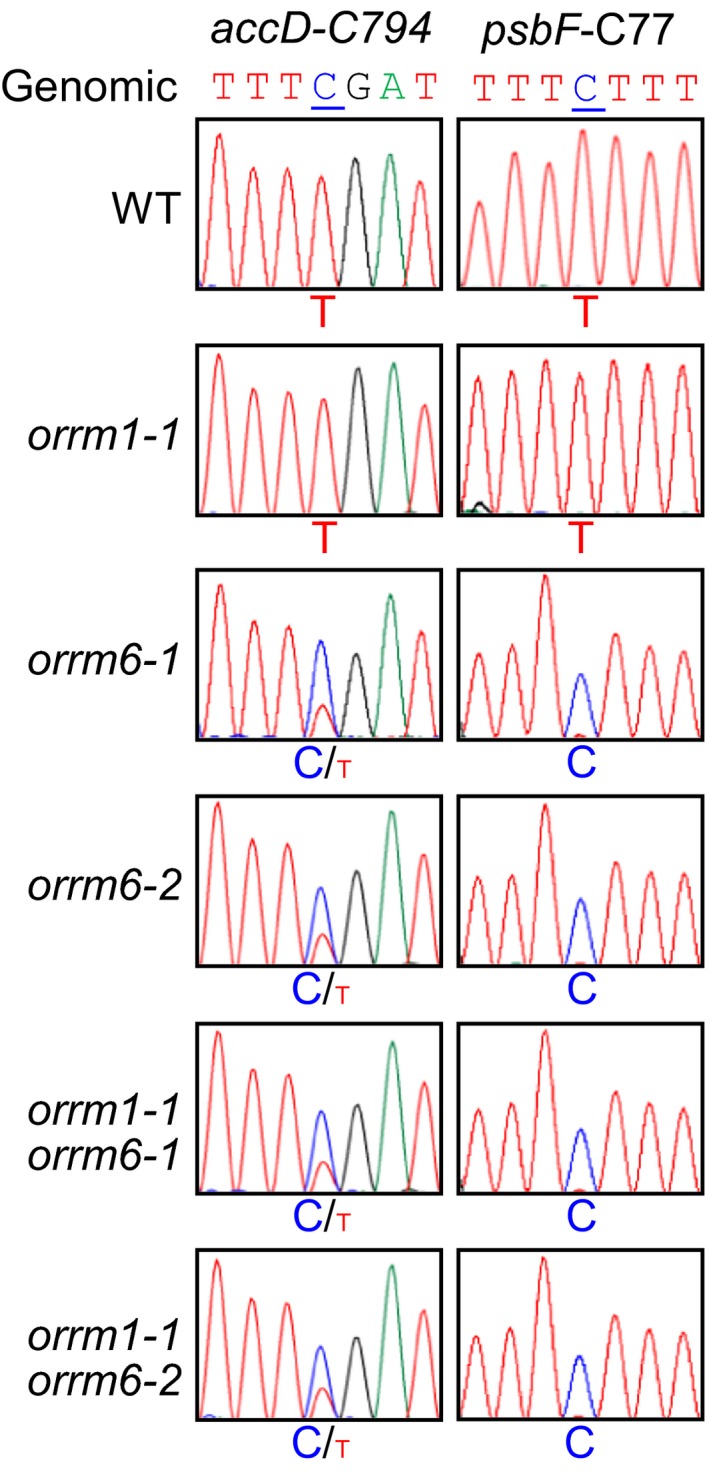
Sanger sequencing of two plastid RNA editing sites affected by loss‐of‐function mutations in *ORRM6*. RT‐PCR products surrounding the editing sites were directly sequenced. The seven‐nucleotide sequences encompassing the cytidine target (underlined) were shown. The corresponding genomic sequences of these sites were displayed as controls

The *orrm6‐1* and *orrm6‐2* single mutants were previously found to show substantial reduction in editing extent at *accD*‐C794 and near‐complete loss of editing at *psbF*‐C77 (Hackett et al., 2017). In this study, these two plastid RNA editing sites displayed similar reduction in editing extent in the *orrm6‐1* and *orrm6‐2* single mutants and the *orrm1‐1 orrm6‐1* and *orrm6‐1 orrm6‐2* double mutants but were unchanged in the *orrm1‐1* single mutant (Figure 4).

**Figure 5:**
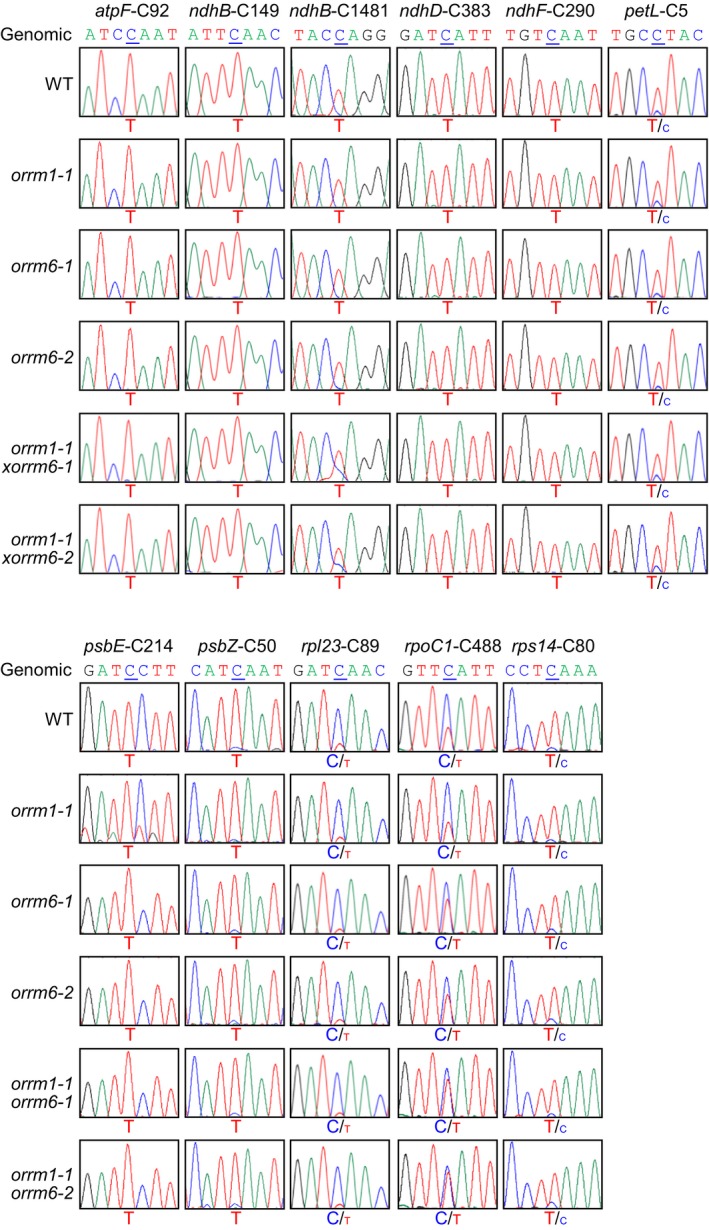
Sanger sequencing of 11 plastid RNA editing sites not affected in *orrm1* or *orrm6* mutants. RT‐PCR products surrounding the editing sites were directly sequenced. The seven‐nucleotide sequences encompassing the cytidine target (underlined) were shown. The corresponding genomic sequences of these sites were displayed as controls

Among the 34 validated plastid RNA editing sites, 11 sites were not affected in either *orrm1* or *orrm6* mutants: *atpF*‐C92, *ndhB*‐C149, *ndhB*‐C1481, *ndhD*‐C383, *ndhF*‐C290, *petL*‐C5, *psbE*‐C214, *psbZ*‐C50, *rpl23*‐C89, *rpoC1*‐C488, and *rps14*‐C80 (Figure 5) (Hackett & Lu, 2017; Hackett et al., 2017; Sun et al., 2013). These 11 sites were not affected in the *orrm1‐1 orrm6‐1* and *orrm1‐1 orrm6‐2* double mutants (Figure 5). Taken together, the total number of plastid RNA editing sites affected in the *orrm1 orrm6* double mutants was the sum of sites affected in the *orrm1* and *orrm6* single mutants. This suggests that simultaneous mutations in the two plastid‐targeted ORRM proteins do not cause additional loss or reduction in editing extent at other plastid RNA editing sites.

## 4 DISCUSSION

The loss‐of‐function mutation in the *ORRM1* and *ORRM6* genes resulted in near‐complete loss or substantial reduction in editing at 21 and two plastid RNA editing sites, respectively (Hackett & Lu, 2017; Hackett et al., 2017; Sun et al., 2013). The 12 plastid RNA editing sites that showed near‐complete loss of editing in the *orrm1* single mutant displayed similar loss of editing in the *orrm1 orrm6* double mutants but were unchanged in the *orrm6* single mutants (Figure 3). This suggests that ORRM1 is the sole ORRM protein at these 12 plastid RNA editing sites. The nine plastid RNA editing sites that showed substantial reduction in editing extent displayed similar reduction in editing extent in the *orrm1 orrm6* double mutants but was unchanged in the *orrm6* single mutants (Figure 3). This suggests that ORRM6 is not responsible for residual editing at these nine plastid RNA editing sites in the *orrm1* single mutant. The *psbF*‐C77 RNA editing site that showed near‐complete loss of editing in the *orrm6* single mutants displayed similar loss of editing in the *orrm1 orrm6* double mutants but was unchanged in the *orrm1* single mutant (Figure 4). This suggests that ORRM6 is the sole ORRM protein at *psbF*‐C77. The *accD*‐C794 RNA editing site that showed substantial reduction in editing extent in the *orrm6* single mutants displayed similar reduction in editing extent in the *orrm1 orrm6* double mutants but was unchanged in the *orrm1* single mutant (Figure 4). This suggests that ORRM1 is not responsible for the residual editing at *accD*‐C794 in the in the *orrm6* single mutants. The 11 plastid RNA editing sites that were not affected in either *orrm1* or *orrm6* mutants remained unchanged in the *orrm1 orrm6* double mutants (Figure 5). This suggests that neither ORRM1 nor ORRM6 functions at these 11 plastid RNA editing sites. Taken together, the results in this study indicate that ORRM1 and ORRM6 are in charge of distinct sets of plastid RNA editing sites and that simultaneous mutations in *ORRM1* and *ORRM6* genes do not cause additional reduction in editing extent at other plastid RNA editing sites. This is consistent with the lack of physical interaction between ORRM1 and ORRM6 proteins in the reciprocal bimolecular fluorescence complementation assay (Hackett et al., 2017).

**Figure 6:**
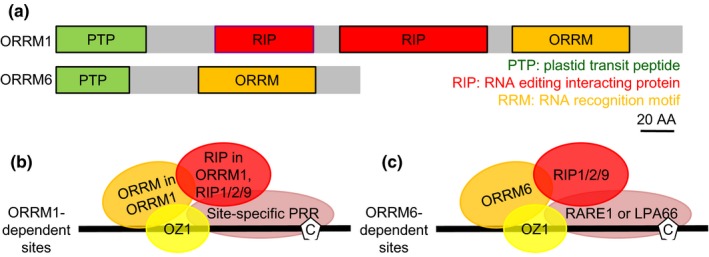
Domain composition of ORRM1 and ORRM6 and models for the editing complexes at the plastid RNA editing sites affected by mutations in *ORRM1* and *ORRM6*. (a) Domain composition of full‐length ORRM1 and ORRM6 proteins. (b) Model for the editing complexes at the 21 ORRM1‐dependent plastid RNA editing sites*: accD*‐C1568, *clpP*‐C559, *matK*‐C640, *ndhB*‐C467, *ndhB*‐C586, *ndhB*‐C746, *ndhB*‐C830, *ndhB*‐C836, *ndhB*‐C872, *ndhB*‐C1255, *ndhD*‐C2, *ndhD*‐C674, *ndhD*‐C878, *ndhD*‐C887, *ndhG*‐C50, *rpoA*‐C200, *rpoB*‐C338, *rpoB*‐C551, *rpoB*‐C2432, *rps12*‐intron, and *rps14*‐C149. (c) Model for the editing complexes at the two ORRM6‐dependent plastid RNA editing sites: *accD*‐C794 and *psbF*‐C77. The PPR protein at *accD*‐C794 and *psbF*‐C77 is RARE1 and LPA66, respectively. For simplicity, only one name is shown for proteins with multiple names (*e.g*., “RIP1” for RIP1/MORF8). Black lines represent transcripts; the letter C in white pentagons represents the cytidine target

ORRM1 was found to interact directly with PPR proteins CRR28 and OTP82, via its duplicated RIP/MORF moiety (Figure 6a) (Sun et al., 2013). CRR28 is necessary for editing at *ndhB*‐467 and *ndhD*‐878 (Okuda et al., 2009), while OTP82 is required for editing at *ndhG*‐50 and *ndhB*‐836 (Okuda et al., 2010). These four plastid RNA editing sites were affected in the *orrm1* single mutant and the *orrm1 orrm6* double mutants (Figure 3). Interestingly, ORRM1 did not appear to interact directly with PPR protein ORGANELLE TRANSCRIPT PROCESSING81 (OTP81), which is required for efficient editing at *accD*‐C1568, *matK*‐C640, *ndhB*‐C872, *rpoB*‐C2432, and *rps12*‐intron (Wagoner et al., 2015). These five plastid RNA editing sites were affected in the *orrm1* single mutant and the *orrm1 orrm6* double mutants (Figure 3). These observations suggest that ORRM1 may interact with some PPR proteins directly via its own RIP/MORF domains, or indirectly associate with other PPR proteins.

ORRM1 was found to interact with plastid‐targeted RIPs/MORFs (Sun et al., 2015). The 21 plastid RNA editing sites affected in the *orrm1* single mutant and the *orrm1 orrm6* double mutants (Figure 3) were also affected in the *rip1*, *rip2*, and/or *rip9* mutants (Bentolila et al., 2012; Takenaka et al., 2012). RIP1/MORF8 was found to interact with the plastid‐targeted PPR protein OTP81 (Wagoner et al., 2015). RIP2/MORF2 and RIP9/MORF9 were found to interact with plastid‐targeted PPR proteins CRR28, DYW1, OTP81, and OTP82 (Wagoner et al., 2015; Zhang et al., 2014). CRR28 is required for editing at *ndhB*‐467 and *ndhD*‐878 (Okuda et al., 2009); DYW1 is essential for editing at *ndhD*‐C2 (Boussardon et al., 2012, 2014); OTP81 is necessary for efficient editing at *accD*‐C1568, *matK*‐C640, *ndhB*‐C872, *rpoB*‐C2432, and *rps12*‐intron (Wagoner et al., 2015); OTP82 is needed for editing at *ndhG*‐50 and *ndhB*‐836 (Okuda et al., 2010). These ten plastid RNA editing sites were substantially affected in the *orrm1* single mutant and the *orrm1 orrm6* double mutants (Figure 3).

Furthermore, ORRM1 was found to interact directly with OZ1 (Sun et al., 2015). The 21 plastid RNA editing sites affected in the *orrm1* single mutant and the *orrm1 orrm6* double mutants (Figure 3) were also affected in the *oz1* mutant (Sun et al., 2015). Therefore, the editing complex at the 21 ORRM1‐dependent plastid RNA editing sites probably contains ORRM1, site‐specific PPR protein(s), RIPs/MORFs (RIP1/MORF8, RIP2/MORF2, and/or RIP9/MORF9), and OZ1 (Figure 6b).

Unlike ORRM1, ORRM6 does not contain any RIP/MORF domains (Figure 6a); therefore, ORRM6 is not expected to interact directly with PPR proteins. Indeed, ORRM6 failed to interact with plastid‐targeted PPR proteins LOW PSII ACCUMULATION66 (LPA66) and RARE1 (Hackett et al., 2017), which are required for editing at *accD*‐C794 and *psbF*‐C77, respectively (Cai et al., 2009; Robbins, Heller, & Hanson, 2009). These two plastid RNA editing sites were substantially affected in the *orrm6* single mutant and the *orrm1 orrm6* double mutants (Figure 4). ORRM6 was found to interact directly with RIP1/MORF8, RIP2/MORF2, RIP9/MORF9, and OZ1 (Hackett et al., 2017). The two ORRM6‐dependent plastid RNA editing sites (*accD*‐C794 and *psbF*‐C77) were also affected in *rip1*, *rip2*, *rip9*, and *oz1* mutants (Sun et al., 2015; Takenaka et al., 2012). Therefore, the editing complex at the two ORRM6‐dependent plastid RNA editing sites probably contains ORRM6, site‐specific PPR protein (*i.e.*, RARE1 for *accD*‐C794 and LPA66 for *psbF*‐C77), RIPs/MORFs (RIP1/MORF8, RIP2/MORF2, and/or RIP9/MORF9), and OZ1 (Figure 6c).

It remains unknown what plastid RRM‐containing protein(s) participate in RNA editing at the 11 plastid RNA editing sites that were not affected in the *orrm1* and *orrm6* single mutants (Figure 5). Two potential candidates are the 31 KD CHLOROPLAST PROTEIN A and B (*i.e.*, CP31A and CP31B). CP31A and CP31B belong to a small group of chloroplast ribonucleoproteins that contain two RRM domains (Tillich et al., 2009). Among these 11 plastid RNA editing sites, five displayed reduced editing extent in *cp31a cp31b* single and/or double mutants: *ndhB*‐C1481, *ndhF*‐C290, *petL*‐C5, *psbZ*‐C50, and *rpoC1*‐C488 (Tillich et al., 2009). Therefore, CP31A and CP31B may serve as the RRM‐containing protein(s) at these five plastid RNA editing sites. However, the editing extent at the other six plastid RNA editing sites (*atpF*‐C92, *ndhB*‐C149, *ndhD*‐C383, *psbE*‐C214, *rpl23*‐C89, and *rps14*‐C80) was not affected by mutations in *CP31A or CP31B*. Further studies are needed to identify the plastid RRM‐containing protein(s) at these plastid RNA editing sites.

## AUTHOR CONTRIBUTIONS

A.M.S., M.B.S., J.P.O., and Y.L. conducted experiments. A.M.S. analyzed data. Y.L. conceived the project, designed and supervised the experiments, and wrote the article.

## Supporting information

Table S1

Table S2

Supplementary Material
