# Supplementary material for: Two organelle RNA recognition motif proteins affect distinct sets of RNA editing sites in the Arabidopsis thaliana plastid
Source: Plant Direct. 2020 Apr 6;4(4):e00213. doi: 10.1002/pld3.213 (PMC7132558; doi:10.1002/pld3.213)
Supplement: Supplementary file 1 — Table S1 [file PLD3-4-e00213-s001.pdf]

Table S1. Primers used in this study

Primer	Sequence	Use
SALK_072648LP	TCACATTTCAAGATTGATGTTTGAC	Left genomic primer for *orrm1-1*
SALK_072648RP	AACCCGAAATGGGTATCAAAG	Right genomic primer for *orrm1-2*
LBa1	TGGTTCACGTAGTGGGCCATCG	Left border primer for SALK lines
SAIL_763_A05LP	AAAATGCAGATTAGGCGGTG	Left genomic primer for *orrm6-1*
SAIL_763_A05RP	CAAAAAGGTTGCAGAAAGCTG	Right genomic primer for *orrm6-1*
LB3	TAGCATCTGAATTTCATAACCAATCTCGATACAC	Left border primer for SAIL lines
WiscDsLox485-488P23LP	GGAAAGCAAAACCTTTTGGTC	Left genomic primer for *orrm6-2*
WiscDsLox485-488P23RP	AGTTTAAGGGGATAGGGGTCC	Right genomic primer for *orrm6-2*
p745	AACGTCCGCAATGTGTTATTAAGTTGTC	Left border primer for WiscDsLox lines
accD_1_F	TTCATTTGTAGTGAAAGCGG	Forward primer for *accD*-C794
accD_1_R	TTTCGCCTACTACGGATCCC	Reverse primer for *accD*-C794
accD_2_F	CTACTACCGGTGGAGTGACAGC	Forward primer for *accD*-C1568
accD_2_R	AGAATCTGATCTAACAACAGGGAA	Reverse primer for *accD*-C1568
atpF_1_F	GAGTTTCGGATTTAATACCG	Forward primer for *atpF*-C92
atpF_1_R	AGCTCCTTGTAAAGCTTGTTG	Reverse primer for *atpF*-C92
clpP_1_F	TTGGGTTGACATATACAACCG	Forward primer for *clpP*-C559
clpP_1_R	TGAACCGCTACAAGATCAAC	Reverse primer for *clpP*-C559
matK_2_F	CGTTACCGGGTAAAAGATGC	Forward primer for *matK*-C640
matK_2_R	AGCGGCGTATCCTTTGTTGC	Reverse primer for *matK*-C640
ndhB_1-3_F	TTTGCTTCTCTTCGATGGAAG	Forward primer for three *ndhB* sites
ndhB_1-3_R	ACGACTGGAGTGGGAGATCCTTC	Reverse primer for three *ndhB* sites
ndhB_7-12_F	CGTATACGAAGGATCTCCCAC	Forward primer for six *ndhB* sites
ndhB_7-12_R	CCTGAGCAATCGCAATAATCG	Reverse primer for six *ndhB* sites
ndhD_1-5_F	TTGAGTACGCGTTCTTTGGAC	Forward primer for five *ndhD* sites
ndhD_1-5_R	AATAGCTCCATTAAGTCCAGG	Reverse primer for five *ndhD* sites
ndhF_2_F	AAAACCTTCGCCGCATGTGG	Forward primer for *ndhF*-C290
ndhF_2_R	GCATTCGCTGCAATAGGTCG	Reverse primer for *ndhF*-C290
ndhG_1_F	ATGGATTTGCCTGGACCAATAC	Forward primer for *ndhG*-C50
ndhG_1_R	TTGATAAATGAATTCCTATTTGTTG	Reverse primer for *ndhG*-C50
petL_2_F	AAATTTGGTAATTAACACGG	Forward primer for *petL*-C5
petL_2_R	ATTTCAATTGAAACTTAGGG	Reverse primer for *petL*-C5
psbE_1_psbF_1_F	ACAGGAGAACGTTCTTTTGC	Forward primer for *psbE*-C214 and *psbF*-C77
psbE_1_psbF_1_R	ATATAATCCATCCGAATGGG	Reverse primer for *psbE*-C214 and *psbF*-C77
psbZ_1_F	ATGAGATACGCGATCCAGTATAC	Forward primer for *psbZ*-C50
psbZ_1_R	TCAAGAGATAAGAGAATTAAGGATAC	Reverse primer for *psbZ*-C50
rpl23_1_F	AAGAGGTGGAATAGAATAACCCG	Forward primer for *rpl23*-C89
rpl23_1_R	CAATTCCTACTGGATGCACGC	Reverse primer for *rpl23*-C89
rpoA_1_F	GTAAGCGTCTTTATTATGGACGC	Forward primer for *rpoA*-C200
rpoA_1_R	CTTGATGAAGTGCTTCTTTAGGAG	Reverse primer for *rpoA*-C200
rpoB_1_3_F	GAAAACCAGTAGGAATATGC	Forward primer for two *rpoB* sites
rpoB_1_3_R	GTCTCCAATTAATATTTCGGCG	Reverse primer for two *rpoB* sites
rpoB_7_F	GAGGTGGGTTCAGAAAAAGG	Forward primer for *rpoB*-C2432
rpoB_7_R	TATCTGTCCTACATTCATGCG	Reverse primer for *rpoB*-C2432
rpoC1_1_F	AGTTTTGTGAACAATGTGGAGTTG	Forward primer for *rpoC1*-C488
rpoC1_1_R	TGAATGATGGGTCTCAACTCGG	Reverse primer for *rpoC1*-C488
rps12_1_F	CTTGTACAATTCACATTCTTTGGC	Forward primer for *rps12*-intron
rps12_1_R	ACAAGACAGCCAATCCGAAAC	Reverse primer for *rps12*-intron
rps14_1-2_F	TTATAGGGAGAAGAAGAGGC	Forward primer for two *rps14* sites
rps14_1-2_R	TACCAGCTTGATCTTGTTGC	Reverse primer for two *rps14* sites
