# Supplementary material for: Two organelle RNA recognition motif proteins affect distinct sets of RNA editing sites in the Arabidopsis thaliana plastid
Source: Plant Direct. 2020 Apr 6;4(4):e00213. doi: 10.1002/pld3.213 (PMC7132558; doi:10.1002/pld3.213)
Supplement: Supplementary file 2 — Table S2 [file PLD3-4-e00213-s002.pdf]

Table S2. Functions of transcripts edited in the Arabidopsis plastid. PE – partially edited (<50%) in the wild type.

Gene function	Biological process	Gene abbreviation	Edited nucleotide	Affected in
β subunit of acetyl-CoA carboxylase	Fatty acid synthesis	*accD*	C794	*orrm6*
		accD	C1568	*orrm1*
F subunit of ATP synthase	Photophosphorylation	*atpF*	C92	
Caseinolytic protease P1	Protein quality control	*clpP*	C559	*orrm1*
Maturase K	RNA splicing	*matK*	C706	*orrm1*
B subunit of NADH dehydrogenase	Cyclic electron flow	*ndhB*	C149	
		ndhB	C467	*orrm1*
		ndhB	C586	*orrm1*
		ndhB	C746	*orrm1*
		ndhB	C830	*orrm1*
		ndhB	C836	*orrm1*
		ndhB	C872	*orrm1*
		ndhB	C1255	*orrm1*
		ndhB	C1481	
D subunit of NADH dehydrogenase	Cyclic electron flow	*ndhD*	$C2^{PE}$	*orrm1*
		ndhD	C383	
		ndhD	C674	*orrm1*
		ndhD	C878	*orrm1*
		ndhD	C887	*orrm1*
F subunit of NADH dehydrogenase	Cyclic electron flow	*ndhF*	$C290^{PE}$	
G subunit of NADH dehydrogenase	Cyclic electron flow	*ndhG*	C50	*orrm1*
L subunit of cytochrome b_6f	Photosynthetic electron transport	*petL*	$C5^{PE}$	
α subunit of cytochrome b_{559} in PSII	Photosynthetic electron transport	*psbE*	C214	
β subunit of cytochrome b_{559} in PSII	Photosynthetic electron transport	*psbF*	C77	*orrm6*
Z subunit of PSII	Photosynthetic electron transport	*psbZ*	C50	
Ribosomal protein L23	Translation	*rpl23*	C89	
α subunit of RNA polymerase	Transcription	*rpoA*	C200	*orrm1*
β subunit of RNA polymerase	Transcription	*rpoB*	C338	*orrm1*
		rpoB	$C551^{PE}$	*orrm1*
		rpoB	C2432	*orrm1*
β′ subunit of RNA polymerase	Transcription	*rpoC1*	$C488^{PE}$	
Ribosomal protein S12	Translation	*rps12-intron*	$C(i1\ 58)^{PE}$	*orrm1*
Ribosomal protein S14	Translation	*rps14*	C80	
		rps14	C149	*orrm1*
