# Supplementary material for: Two organelle RNA recognition motif proteins affect distinct sets of RNA editing sites in the Arabidopsis thaliana plastid
Source: Plant Direct. 2020 Apr 6;4(4):e00213. doi: 10.1002/pld3.213 (PMC7132558; doi:10.1002/pld3.213)
Supplement: Supplementary file 3 — Supplementary Material [file PLD3-4-e00213-s003.pdf]

Reviewer #1:

This manuscript describes an analysis of an Arabidopsis double mutant lacking two members of the 'ORRM' family of organelle RNA editing factors (ORRM1 and ORRM6). Previously, the phenotypes and editing defects of single orrm1 and orrm6 mutants were reported. The new contribution here is the analysis of a double mutant. The finding is that the editing defects in the double mutant are additive, as expected if each factor is involved in specifying editing of different sites.

Given what is known about organellar RNA editing in general and ORRM proteins in particular, this outcome is what would be expected. The only scenario I can think of in which this would not be the case would be one in which ORRM1 and ORRM6 have functional redundancy (i.e., either one or the other could act at certain sites, such that no phenotype is observed in single mutants). I am not aware of this ever being observed in organellar RNA editing mutants to date, and this possibility is not mentioned in the paper. As such, the motivation for analyzing the double mutant is unclear.

Although I don't doubt that the results are correct, this manuscript describes just one experiment of unclear motivation, and the results are as expected.

Response: Thank you for your comments. We revised the last paragraph of the Introduction (line 136 – 152 in the changes-tracked version; line 134 – 150 in the clean version) to describe the motivation for analyzing the double mutants:

"As mentioned above, the *orrm1-1* single mutant showed substantial reduction in editing extent at nine plastid RNA editing sites (Sun et al., 2013). This begs the question whether other plastid-targeted ORRM or ORRM-like protein(s), such as ORRM6, is responsible for residual editing at these nine plastid RNA editing sites in the *orrm1-1* single mutant. The orrm6 single mutants displayed substantial reduction in editing extent at accD-C794 (Hackett et al., 2017; Hackett and Lu, 2017). This raises the question whether other plastid-targeted ORRM or ORRM-like protein(s), such as ORRM1, is responsible for residual editing at accD-C794 in the *orrm6* single mutants. Furthermore, recombinant ORRM6 protein showed some binding activity towards the synthetic RNA flanking *psbE*-C214, a plastid RNA editing site not affected by the loss-of-function mutations in the *ORRM6* gene (Hackett et al., 2017). This made us consider whether ORRM6 could function at additional plastid RNA editing sites that are not identified by loss-of-function mutations in the *ORRM6* gene. To investigate the functional relationship between the two plastid-targeted ORRM proteins and explore the possible existence of ORRM-like proteins in the Arabidopsis plastid, we generated *orrm1 orrm6* double homozygous Arabidopsis mutants, examined their plastid RNA editing pattern, perform a series of morphological and physiological analyses, and compared them with the wild type and the single mutants. The results showed that ORRM1 and ORRM6 are in charge of distinct sets of plastid RNA editing sites."

Minor points:
The writing needs editing for grammar, etc. This is apparent with the first sentence of the introduction.

Response: Thank you for your comments. We carefully edited the manuscript to eliminate grammatically problems. For example the first sentence of the Introduction (line 49 – 50 in the changes-tracked version; line 49 – 50 in the clean version) was changed to:

"RNA editing is a post-transcriptional process through which discrete changes are introduced to RNA sequences."

The experiments include analyses of chlorophyll and carotenoid content, and a fluorescence readout of PSII activity. Given that the effects on pigments and photosynthesis all derive from the RNA editing defects, the motivation for measuring pigment and photosynthesis was unclear.

Response: Thank you for your comments. We included the following sentences in the Results (line 208 – 222 in the changes-tracked version; line 206 – 219 in the clean version), to address the motivation for measuring pigment and photosynthesis:

"As reported in a previous study (Sun et al., 2013), the *orrm1-1* mutant did not show any phenotypic defect, presumably because none of the plastid RNA transcripts affected in the *orrm1-1* mutant is essential. The *orrm1-1* mutant was actually slightly bigger than the Columbia wild type. However, it is not clear whether loss-of-function mutation in the *ORRM1* gene causes changes in pigment contents and photosynthetic efficiency. The *orrm6-1* and *orrm6-2* mutants were substantially smaller than the wild type and they displayed reduced PSII photochemical efficiency, small and pale green leaves, and stunted growth (Hackett et al., 2017), presumably because *psbF*, one of the two plastid RNA transcripts affected in the *orrm6* mutants, encodes an essential PSII subunit. To examine whether loss-of-function mutation in the *ORRM1* gene causes changes in fresh weights, leaf numbers, pigment contents, and photosynthetic efficiency and whether simultaneous loss-of-function mutations in *ORRM1* and *ORRM6* genes result in additive effects, we compared phenotypes, measured fresh weights of the above-ground portion of the plants, counted leaf numbers, determined pigment contents, and measured photosynthetic parameters in four-week-old wild type, *orrm1* and *orrm6* single and double mutants."

The discussion is quite long given the nature of the experiment and result. Much of it revolves around the specific editing defects in the orrm1 and orrm6 mutants. Most of these things were already discussed in the context of the papers on the single mutants. The discussion should focus on new insights from the experiment performed here - the analysis of the double mutant. As such, it should be quite short.

Response: Thank you for your comments and suggestions. We expanded the paragraph (see line 299 – 328 in the changes-tracked version; line 296 – 320 in the clean version; and below) discussing new insights from experiments performed in this study, i.e., double mutant analyses.

"The loss-of-function mutation in the *ORRM1* and *ORRM6* genes resulted in near-complete loss or substantial reduction in editing at 21 and two plastid RNA editing sites, respectively (Sun et al., 2013; Hackett et al., 2017; Hackett and Lu, 2017). The 12 plastid RNA editing sites that showed near-complete loss of editing in the *orrm1* single mutant displayed similar loss of editing in the *orrm1 orrm6* double mutants but were unchanged in the *orrm6* single mutants (Figure 3). This suggests that ORRM1 is the sole ORRM protein at these 12 plastid RNA editing sites. The nine plastid RNA editing sites that showed

substantial reduction in editing extent displayed similar reduction in editing extent in the *orrm1 orrm6* double mutants but were unchanged in the *orrm6* single mutants (Figure 3). This suggests that ORRM6 is not responsible for residual editing at these nine plastid RNA editing sites in the orrm1 single mutant. The *psbF*-C77 RNA editing site that showed near-complete loss of editing in the *orrm6* single mutants displayed similar loss of editing in the *orrm1 orrm6* double mutants but were unchanged in the *orrm1* single mutant (Figure 4). This suggests that ORRM6 is the sole ORRM protein at *psbF*-C77. The *accD*-C794 RNA editing site that showed substantial reduction in editing extent in the *orrm6* single mutants displayed similar reduction in editing extent in the *orrm1 orrm6* double mutants but were unchanged in the *orrm1* single mutant (Figure 4). This suggests that ORRM1 is not responsible for the residual editing at *accD*-C794 in the in the *orrm6* single mutants. The 11 plastid RNA editing sites that were not affected in either *orrm1* or *orrm6* mutants remained unchanged in the *orrm1 orrm6* double mutants (Figure 5). This suggests that neither ORRM1 nor ORRM6 functions at these 11 plastid RNA editing sites. Taken together, the results in this study indicate that ORRM1 and ORRM6 are in charge of distinct sets of plastid RNA editing sites and that simultaneous mutations in *ORRM1* and *ORRM6* genes do not cause additional reduction in editing extent at other plastid RNA editing sites. This is consistent with the lack of physical interaction between ORRM1 and ORRM6 proteins in the reciprocal bimolecular fluorescence complementation assay (Hackett et al., 2017)."

Results from the double mutant analyses enable us to have a better picture of the editing complexes at ORRM1- and ORRM6-dependent plastid RNA editing sites. We shortened related paragraphs (line 329 – 391 in the changes-tracked version; line 321 – 374 in the clean version) to match the nature of the experiments and results in this study.

Reviewer #2:

In this manuscript, Searing et al. provide an analysis of Arabidopsis single and double mutants in the ORRM1 and ORRM6 genes encoding proteins controlling editing sites in the plastid. While each single mutant was known to be affected for distinct editing sites in plastid-encoded transcripts, they report here that the double mutant does not display any additional reduction in editing than the combination of editing defects corresponding to each single mutant. Phenotypically, they also document that the double orrm1 orrm6 mutant cannot be distinguished from the single orrm6, which is affected for growth due to loss of editing sites in transcripts encoding proteins required for photosynthesis. The study is carefully executed and the data are well presented. The manuscript is very well written and the authors have done a very good job articulating the Science. Below are my comments for improvement of the manuscript.

1) The authors mentioned that the orrm1-1 mutant appears bigger than the wild-type. This appears also to be the case in the first description of the mutant (Sun et al., 2013). This is an interesting observation that is possibly linked to loss of editing at sites controlled by ORRM1. I am wondering if the authors can provide additional information documenting the phenotype by including measurements or a description of some anatomical traits (for example stem size, etc…)? Is the mutant truly bigger or is more advanced in its development than the wild-type?

Response: Thank you for your suggestions. We measured the fresh weight of the above-ground portion of the plants and counted the number of leaves in four-week-old plants. The results showed that the four-week-old *orrm1-1* mutant had a significantly heavier fresh weight (Figure 1b) and a

significantly larger leaf number (Figure 1c) than the wild type grown at the same time under the same conditions. This suggests that the *orrm1-1* mutant is truly bigger and possibly more advanced in its development than the wild type. We described this finding in the Results (line 226 - 229 in the changes-tracked version; line 223 – 226 in the clean version).

2) Based on figure 1, I am also not entirely convinced the orrm1 orrm6 mutant is phenotypically similar to the orrm6 mutant. So including additional description of the phenotype might solidify the authors' statement (see my comment above).

Response: Thank you for your suggestions. We measured the fresh weight of the above-ground portion of the plants and counted the number of leaves in four-week-old plants. The results showed that four-week-old *orrm1 orrm6* double mutants had statistically similar fresh weights (Figure 1b) and statistically similar leaf numbers (Figure 1c) as the *orrm6* single mutants grown at the same time under the same conditions. This suggests that the *orrm1 orrm6* double mutants are indeed phenotypically similar to the *orrm6* single mutants. We described this finding in the Results (line 229 - 233 in the changes-tracked version; line 226 – 230 in the clean version).

3) It would be informative to the readership to know what phenotypical consequence might be expected due to the loss of editing in the different transcripts examined in this study. For instance, it appears most of the sites controlled by ORRM1 are in transcripts encoding subunits of the plastid NADH dehydrogenase complex. Maybe a table including the function the edited transcripts are controlling would be useful to the reader who might not be that familiar with plastid-encoded gene products.

Response: Thank you for your suggestions. We included "Table S2. Functions of transcripts edited in the Arabidopsis plastid" in the manuscript." In this table, we list the full names and biological processes of transcripts edited in the Arabidopsis plastid.

4) This is a minor comment but I think the term "higher plants", although used, is not very precise. It is best to use the term vascular plants if this is what the authors were referring to in their abstract.

Response: Thank you for your suggestions. We changed "higher plants" to "vascular plants" in the Abstract (line 24 in the changes-tracked version; line 24 in the clean version).
